# Compilation of 10 Years of MIRU-VNTR Data: Canadian National Tuberculosis Laboratory's Experience

**DOI:** 10.1155/2022/3505142

**Published:** 2022-08-22

**Authors:** Meenu K. Sharma, Debra Janella, Alisa McGurran, Cindi Corbett, Heather Adam, Pierre-Marie Akochy, David Haldane, Hope MacKenzie, Jessica Minion, Robert Needle, Caroline Newberry, Michael Patterson, Inna Sekirov, Gregory Tyrrell, Hafid Soualhine

**Affiliations:** ^1^National Reference Centre for Mycobacteriology, National Microbiology Laboratory, Public Health Agency of Canada, Winnipeg, Canada; ^2^Department of Medical Microbiology, University of Manitoba, Winnipeg, Canada; ^3^Shared Health, Winnipeg, Manitoba, Canada; ^4^Laboratoire de Sante Publique du Quebec, Montreal, Quebec, Canada; ^5^Public Health Laboratory Network, Halifax, Nova Scotia, Canada; ^6^Atlantic Health Sciences Corporation, Saint John, New Brunswick, Canada; ^7^Saskatchewan Health Authority, Saskatoon, Saskatchewan, Canada; ^8^Newfoundland and Labrador Public Health Laboratory, Saint John, Newfoundland, Canada; ^9^Office of Chief Public Health Officer, Yellowknife, Northwest Territories, Canada; ^10^Government of Nunavut, Iqaluit, Nunavut, Canada; ^11^BC Centre for Disease Control, Vancouver, British Columbia, Canada; ^12^Alberta Health Services, Edmonton, Alberta, Canada

## Abstract

Tuberculosis is a significant cause of morbidity worldwide and is a priority at the provincial and federal levels in Canada. It is known that tuberculosis transmission networks are complex and span many years as well as different jurisdictions and countries. MIRU-VNTR is a universal tuberculosis genotyping method that utilizes a 24-loci pattern and it has shown promise in identifying inter and intrajurisdictional clusters within Canada. MIRU-VNTR data collected over 10 years from the National Reference Centre for Mycobacteriology (NRCM) were analyzed in this study. Some clusters were unique to a single province/territory, while others spanned multiple provinces and/or territories in Canada. The use of a universal laboratory test can enhance contact tracing, provide geographical information on circulating genotypes, and hence, aid in tuberculosis investigation by public health. The housing of all data on one platform, technical ease of the method, easy exchange of data between jurisdictions, and strong collaboration with laboratories and surveillance units at the provincial and federal levels have the potential to identify possible outbreaks in real time.

## 1. Introduction

In 2019, *Mycobacterium tuberculosis* caused disease in an estimated 10 million people worldwide, resulting in 1.2 million and 208,000 deaths from tuberculosis among HIV-negative and HIV-positive individuals, respectively [1]. At the country level, tuberculosis incidence rates vary from <5 to >500 cases per 100,000 persons every year, averaging globally at 130 cases per 100,000 population. The majority of tuberculosis cases occur in African, Southeast Asian, and the Western Pacific regions, representing 25%, 44%, and 18% of global cases, respectively, while Europe and the Americas harbor only 2.5% and 2.9% of global tuberculosis cases [1]. In Canada, the rate of active tuberculosis was 4.9 per 100,000 persons in 2017. More specifically, foreign born cases from tuberculosis endemic regions (14.7 cases per 100,000 persons), Canadian born Indigenous (21.5 per 100,000 persons), and Canadian born nonindigenous (0.5 per 100,000) cases constituted approximately 71.8%, 17.4%, and 7% of total Canadian cases, respectively, in 2017 [2]. Although Indigenous populations consist of 4.9% of the total Canadian population, according to Canada's 2016 Census of Population (Annual Report to Parliament 2020), they constitute 17.4% of annual tuberculosis cases (with incidence rates ranging from 3.5 to 205.8 cases per 100,000 in subsets) [2].

Transmission dynamics of tuberculosis are complicated as the disease can be active, or remain latent for many years in infected individuals. Latent disease may take years before it becomes symptomatic and hence the direction of transmission of tuberculosis is not easy to investigate [3–5]. Tuberculosis transmission across countries or continents increases the complexity of the investigations. In Canada, tuberculosis outbreaks in northern communities have lasted decades due to ongoing transmission or circulation of clonal strains [6], making tuberculosis transmission networks difficult to understand. Over the years, laboratory evidence of tuberculosis transmission has simplified some assessments, but evolution in the methodology of tuberculosis genotyping has introduced additional complexities [7–11].

Genotyping can be utilized in outbreak investigations, deciphering chains of transmission, distinguishing cases of relapse or reinfection, performing surveillance, determining evolutionary trees, taxonomy, and assessment of contamination events. Whole-genome sequencing (WGS) has been an asset in the identification, antimicrobial susceptibility prediction, genotyping, and lineage determination of *M. tuberculosis* isolates [12–17], but these methods remain complicated due to nonstandardized bioinformatics analyses, genomic pipelines, sample types and technical concerns related to mycobacterial DNA extraction and next-generation sequencing technologies [18–21]. While WGS is a powerful and impactful tool, historical genotyping databases for *M. tuberculosis* still utilize the Mycobacterial Interspersed Repetitive Unit—Variable Number Tandem Repeat (MIRU-VNTR) MLVA (Multiple Locus VNTR Analysis) scheme specific for tuberculosis genotyping [11]. A harmonized and reliable PCR-based method of tuberculosis genotyping using MIRU-VNTR can determine the size and repeated number of units in 24 different loci. This method changed tuberculosis genotyping on a global scale as it was reproducible, fast, and high-throughput [10, 22, 23]. MIRU-VNTR patterns can be easily searched in international databases [24, 25]. The MIRU-VNTR 24-loci method was implemented in Canada in 2008 as it is a portable form of data that is not subject to interpretive errors [7, 26] and could genotype *M. tuberculosis* isolates without having to sacrifice discriminatory power [27]. In Canada, federal and provincial tuberculosis programs are indirectly connected [7, 28]. *M. tuberculosis* isolates were sent voluntarily to the federal laboratory for genotyping and the volume of testing was dependent on the testing requirements of the individual provinces/territories. During the study period, all isolates submitted by provinces/territories (except for Ontario) were genotyped and maintained in a federal database at the NRCM in Winnipeg, Canada. To demonstrate the utility of tuberculosis genotyping using a universal method, a 10-year compilation of 24-loci MIRU-VNTR in Canada's NRCM was completed. It is intended to provide information on the predominant clusters in Canada, intra or interprovincial MIRU-VNTR patterns, and evidence of endemic or shared MIRU-VNTR patterns for *M. tuberculosis* in Canada from 2008 to 2017.

## 2. Materials and Methods

### 2.1. Study Sample

Since 2008, MIRU-VNTR genotyping for 24-loci has been routinely performed on all *M. tuberculosis* cultures submitted by provincial tuberculosis laboratories to the NRCM at the National Microbiology Laboratory in Winnipeg. The NRCM database was searched for cultures submitted for MIRU-VNTR between January 1, 2008, and December 31, 2017. A total of 6755 *M. tuberculosis* isolates were received for *M. tuberculosis* genotyping in the 10-year study period. Date of submission, province of submission, MIRU-VNTR 24-digit pattern, and cluster number assignments by *Bionumerics software v7.6.2* were extracted from the database.

### 2.2. MIRU-VNTR

Crude DNA was extracted by boiling 250–500 *μ*l of culture and silica beads for 10 mins followed by 15 min sonication at 35 kHz, as previously described [26]. Genotyping was performed using the 24 locus MIRU-VNTR genotyping Technical Guide [11, 24].

### 2.3. Data Analysis

As previously described, ABI 3130 XL genetic analyzer (Applied Biosystems, CA, USA) was used to obtain data, *GeneMarker v1.*4 was used for data analysis (Softgenetics, PA, USA) and the numerical values were assigned to alleles [11, 26]. UPGMA or Unweighted Pair Group Method with Arithmetic mean phylogenetic analysis was performed using the *Bionumerics software V.7.6.2* on the locally housed database.

### 2.4. Cluster Definitions

In this study, a cluster was defined as containing two or more isolates with an identical 24-loci MIRU-VNTR pattern. A cluster generating algorithm within *BioNumerics software v7.6.2* was used to assign arbitrary cluster numbers to all MIRU patterns. A “cluster alert” was based on the formula to detect the spike of a MIRU pattern in 3 years. If the number of isolates in the most recent year (*Y*3) were more than the number of average isolates in the preceding 2 years (average of *Y*1 + *Y*2), it was flagged as an alert. It helped examine years that were more significant for certain clusters and indicated warning or progression towards an outbreak, lab contamination, or another reason that required further investigation.

(1) $$\begin{array}{c} \text{Cluster alert}=\text{cases of cluster }X\text{ in}Y3>\frac{\left(Y1+Y2\right)}{2}. \end{array}$$

### 2.5. *Mycobacterium tuberculosis* Complex Lineage Prediction by MIRU-VNTR*plus*

For major clusters, MIRU-VNTR patterns were searched within the MIRU-VNTR*plus* database which contains a collection of 186 *M. tuberculosis* lineages. This database has a universal nomenclature for different MLVA MtbC15 and 9; combination based on the 15 loci discriminatory subset (23914 types of MLVA MtbC15 that includes 424, 577, 580, 802, 960, 1644, 1955, 2163b, 2165, 2401, 2996, 3192, 3690, 4052, 415) and a type based on the nine auxiliary loci (2090 types of MLVA MtbC9 that includes 154, 2059, 2347, 2461, 2531, 2687, 3007, 3171, 4348). A phylogenetic tree based on a distance matrix was generated using UPGMA or neighbor-joining clustering algorithms. Strain type (ST) is a numerical code that can be used to further identify MIRU-VNTR lineage [24, 25].

## 3. Results

**Figure 1:**
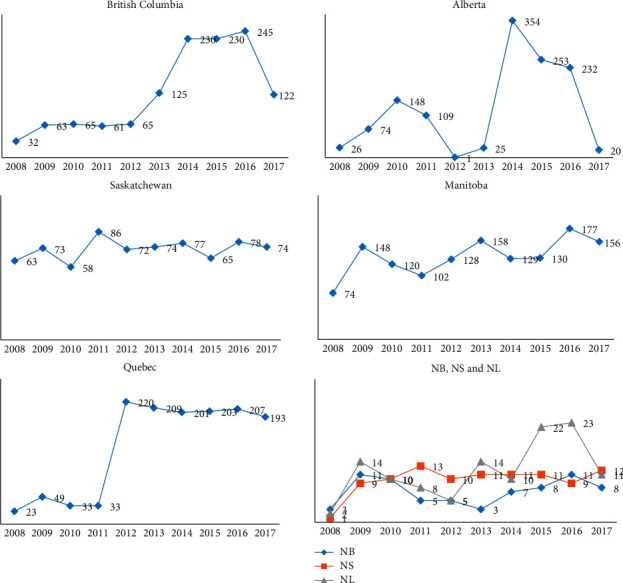
Number of MIRU-VNTR tests reported by the NRCM to various Canadian provinces over the period of 10 years between 2008 and 2017 NB, New Brunswick; NS, Nova Scotia including Prince Edward Island; NL, Newfoundland. Labrador *y* = number of MIRU-VNTR tests reported by the NRCM; *x* = year of submission.

**Figure 2:**
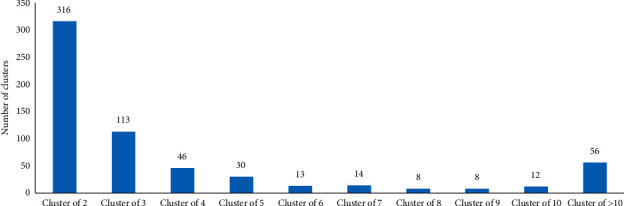
MIRU-VNTR clusters in the NRCM database: data shown are for “two isolates per cluster” to “≥10 isolates per cluster.” A total number of clusters in the database that had identical MIRU-VNTR are also shown in figure. *X* = number of MIRU-VNTR matches (a cluster can be comprised of two to ≥10 isolates); *Y* = number of clusters.

**Figure 3:**
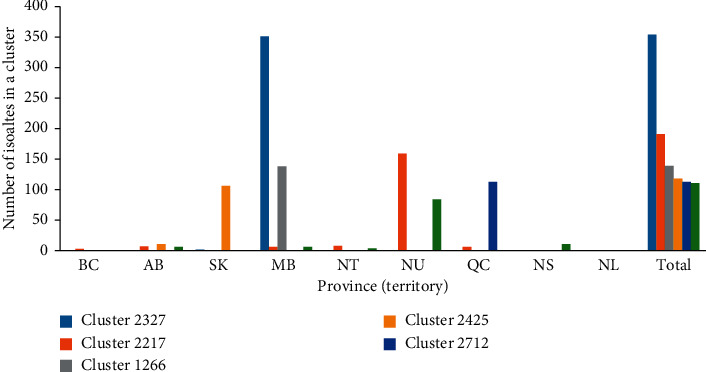
Distribution of six largest MIRU-VNTR clusters that contain more than 100 isolates each, shown per Canadian province or territory. BC, British Columbia; AB, Alberta; SK, Saskatchewan; MB, Manitoba; NT, Northwest Territories; NU, Nunavut; QC, Quebec; NS, Nova Scotia including Prince Edward Island isolates; NL, Newfoundland.

**Figure 4:**
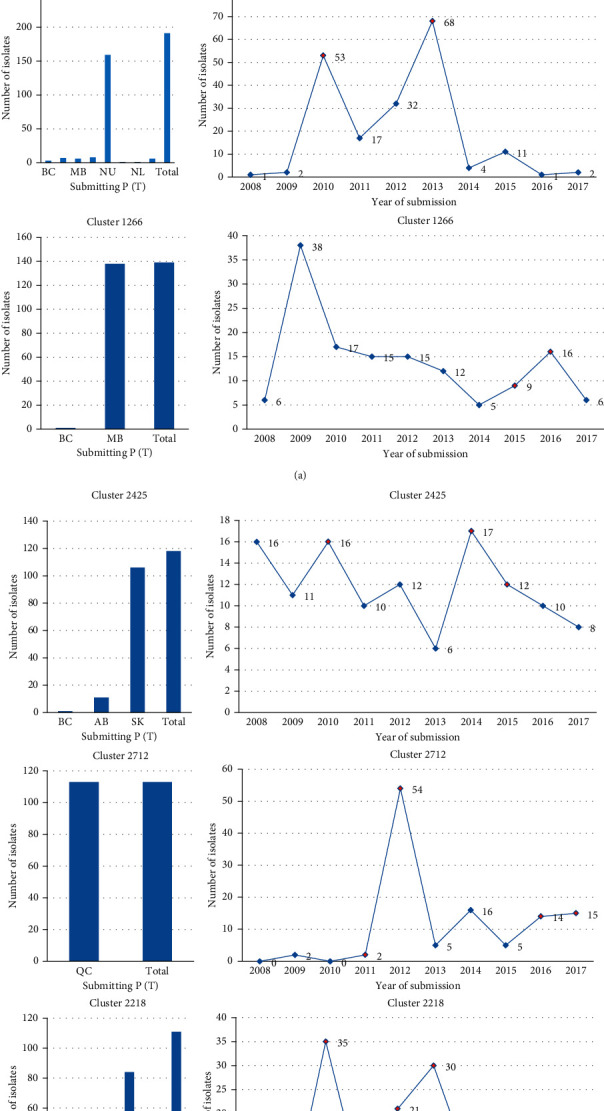
Distribution of the largest six MIRU-VNTR clusters in national laboratory, Canada.

**Figure 5:**
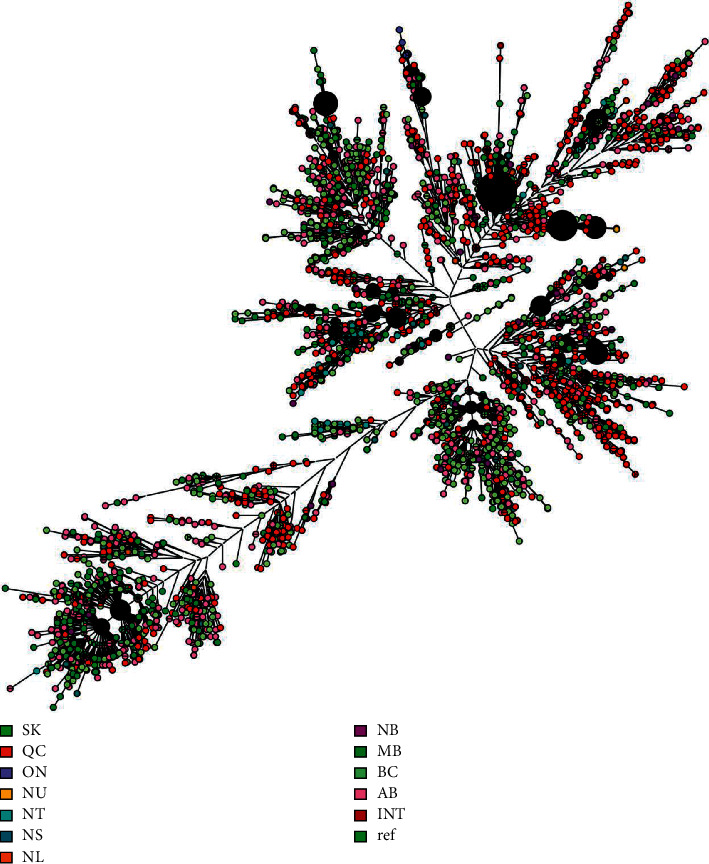
A dendrogram of all MIRU-VNTR patterns and clusters in the NRCM database^*∗*^. ^*∗*^ON (Ontario) submissions reflect submission by parks Canada and not ON provincial TB laboratory. The distance was calculated using the UPGMA clustering algorithm, with different colors representing different provinces or territories. The figure is showing that most patterns are not unique to one jurisdiction. AB, Alberta; BC, British Columbia; MB, Manitoba; INT, International; NT, Northwest Territories; NU, Nunavut; QC, Quebec; NS, Nova Scotia including Prince Edward Island cases; NL, Newfoundland; SK, Saskatchewan.

A 10-year snapshot of MIRU-VNTR data compilation at the NRCM showed that between January 1, 2008, and December 31, 2017, a total of 6755 isolates were received by the NRCM for MIRU-VNTR genotyping, of which 6741 were from Canadian laboratories. Total submissions per year are shown in Figure 1. During these 10 years, a total of 3040 MIRU-VNTR clusters were observed. A total of 2424 isolates had unique MIRU-VNTR patterns and 4331 isolates clustered in 616 MIRU-VNTR patterns. MIRU-VNTR clustering and distribution of clusters in the NRCM database are shown in Figures 2 and 3. The annual distribution of the most common six MIRU-VNTR clusters is presented in Figure 4. A dendrogram of all MIRU-VNTR patterns and clusters in the NRCM database was created using the UPGMA clustering algorithm in *Bionumerics*, which is shown in Figure 5.

The NRCM database showed that there was a steady increase in testing requests from the year 2008–2017 (Figure 1). Before 2008, Manitoba, Saskatchewan, and the Atlantic provinces submitted all culture-positive cases (one isolate per patient) for tuberculosis genotyping. The other provincial laboratories requested tuberculosis genotyping when Public Health departments asked for specific genotyping results or a case was linked with a public health or laboratory investigation. Between the years 2012 and 2013, British Columbia, Alberta, and Quebec submitted all their culture-positive cases (one isolate per patient). A decline in requests in 2016 resulted from the technology transfer of MIRU-VNTR from federal to provincial laboratories, notably to British Columbia and Alberta laboratories. In 2015, retrospective years' isolates from the Northwest Territories were received to build their territorial database by the Alberta laboratory. Accordingly, the Alberta data may be skewed in that year.

The three most predominant MIRU-VNTR clusters in the provinces of Alberta, British Columbia, Saskatchewan, Manitoba, Quebec, and Atlantic provinces are presented in Supplementary Figures S1-S6. These figures show distribution by year of submission date as well as other provinces that share a specific MIRU-VNTR pattern. Graphs showing trends of the largest three clusters within a province are also shown.

A search was conducted within the MIRU-VNTR*plus* database for the largest six clusters contained in the national database and the results are displayed in Table 1. Top MLVA MtbC15-19 matches, the branch distance from the closest match, SpolDB4 strain type (ST) match, the corresponding lineage match (ST), and the closest match from a neighbor-joining phylogenetic tree generated from MIRU-VNTR*plus* are also listed in the table. The MIRU-VNTR*plus* phylogenetic tree with the largest six NRCM clusters is shown in Supplementary Figure S7.

## 4. Discussion

Molecular typing of *M. tuberculosis* is an important tool in contact tracing, investigation of an ongoing outbreak, or investigating false clusters such as laboratory contamination. The 24-loci MIRU-VNTR method improved the discriminatory index of clusters compared with previous methods, such as 12-loci MIRU-VNTR and spoligotyping [11, 29, 30]. The MIRU-VNTR*plus* database can also be used to identify strain types, MIRU-VNTR lineage, correlate to all known global lineages [24, 25], and generate a phylogenetic tree. The rapid turn-around times, high discriminatory power, numerical data, simplified data exchange, and few interpretive errors made MIRU-VNTR an ideal system for genotyping of *M. tuberculosis* in Canada. The public health surveillance system remains the most impactful piece of tuberculosis control as it can utilize laboratory genotyping data and correlate it with case data to interpret tuberculosis chains of transmission within a network [8, 21, 23, 28, 31]. Making the distinction between reinfection and relapse using genotyping has important implications for epidemiological investigations [1].

There are some limitations to this study dataset: (i) The total number of isolates does not reflect the number of cases per province/territory per year as not all jurisdictions submit every culture to the NRCM for tuberculosis genotyping. (ii) Isolates received in batches as part of public health investigations do not reflect the year of case detection. (iii) Some territorial samples may be submitted and reported via provincial laboratories and the NRCM is not always notified of the true geographical origin of the sample. (iv) An additional limitation of MIRU-VNTR is that it does not provide adequate discrimination in clonal outbreaks. (v) Data collected are not in real time.

Of the total 6755 clinical isolates genotyped in the 10-year study period, approximately, one-third of isolates (*n* = 2424) were associated with unique MIRU-VNTR patterns while approximately two-thirds of isolates (*n* = 4331) were associated with 616 different MIRU-VNTR patterns. This demonstrates a large *M. tuberculosis* strain diversity in Canada. The distribution of all clusters is shown in Figure 2. Most MIRU-VNTR clusters were small, for example, 316 clusters were composed of two isolates with 100% MIRU-VNTR identity. There were 56 clusters made up of more than 10 isolates each. Of these, 41 were smaller clusters comprising 11–50 isolates each, nine clusters contained 51–100 isolates each, and six clusters had more than 100 isolates each. These large clusters help provide an in-depth understanding of possible transmissions within each network (Figure 3). Cluster 2327 is predominantly from Manitoba and is the largest cluster to date in Canada. The other five large clusters, with more than 100 isolates each, are similar in size to each other. Two of these clusters are predominantly from Nunavut, and one cluster each is predominantly from Manitoba, Saskatchewan, and Quebec, respectively. Notably, although each of these major clusters is seen predominantly in one province/territory, they span other jurisdictions likely due to the mobility of populations.

As evident from the cluster trends (Figure 4), knowing a baseline for each cluster is crucial. Without critically examining each cluster and its trend, it is difficult to know which clusters were causing an outbreak and which ones were contributing to its baseline. Although both trends are relevant to understanding old or ongoing outbreaks, a spike in cases is a concern for the resurging of an ongoing outbreak or a potential new outbreak. For this study, a “cluster alert” was defined based on our mathematical formula that was calculated using the number of isolates during 3 years. The alert highlights years that were significant for certain clusters. Cluster alert functionality, and algorithms that are flexible and based on a mathematical formula can be adjusted with time and as methodology evolves.

The graphs in Figure 4 show the distribution of the six larger clusters in Canada that have more than 100 isolates each, further distributed by province and year of submission between 2008 and 2017. The data demonstrate that some clusters are endemic to certain jurisdictions while others span multiple jurisdictions. The cluster trends over 10 years may show spiking or declining rates. Cluster 2327 is the largest in the NRCM database, comprising 354 isolates during the study period, and is primarily seen in Manitoba except for one isolate in Alberta and two in Saskatchewan. Compared with other clusters, the baseline/threshold is highest for this cluster. Based on the *cluster alert* definition for this study, the years 2011, 2012, 2013, and 2016 would have been flagged, and hence, would be worthy of further assessment by public health departments. Cluster 2217 is the second-largest cluster in the NRCM database, comprising 191 isolates. It is distributed across many jurisdictions in Canada but the majority is endemic to the Nunavut region. As submissions from Nunavut were batched, critical analysis by the year of submission is not advised. Cluster 1266 is the third largest cluster in the NRCM database, comprising 139 isolates. It is endemic to Manitoba except for one isolate in British Columbia. The years 2015 and 2016 would have been flagged as an *alert* for this cluster. Cluster 2425 is comprised of 139 isolates. It is primarily seen in Saskatchewan with one isolate in British Columbia and 11 isolates in Alberta. As per our analysis criterion, the years 2010, 2014, and 2015 would be flagged for this cluster. Cluster 2712 is comprised of 113 isolates from Quebec. As per our cluster alert definition, the years 2011, 2012, 2016, and 2017 would be flagged with an alert. The data for this cluster are skewed, as retrospective isolates were sometimes sent for genotyping due to an ongoing public health investigation. Cluster 2218 is comprised of 111 isolates. A total of 84 isolates are from Nunavut, along with six from Alberta, six from Manitoba, four from the Northwest Territories, and one from Quebec. The years 2010, 2012, and 2013 would be flagged with alerts for this cluster. If samples for *M. tuberculosis* are routinely genotyped prospectively, cluster trends can be monitored in real time which can impact ongoing outbreak investigations [31]. However, as previously mentioned, various provinces forwarded their cultures to the NRCM for retrospective genotyping and submission dates to NRCM do not correlate with the year of active case detection. Few clusters described in this study were reported in different provinces or territories, as every jurisdiction manages its program and there is no direct communication between different jurisdictions. Additionally, the provincial Personal Health Information Acts limit data exchange. Better communication platforms between provincial and federal as well as laboratory and public health units are needed to allow easier data exchange for tuberculosis disease surveillance.

Eleven Distinct MIRU-VNTR patterns were submitted to the MIRU-VNTR*plus* database. Only two MLVA MtbC15 patterns had a 100% match, but all isolates had an MLVA MtbC19 match in the database. Although the closest lineage match is useful to understand the probable origin of the strains, SpolDB4 ST matches helped provide additional discriminatory information. Clusters belonging to EAI, *X*, Haarlem, LAM, Cameroon, Delhi/CAS, S, and Uganda lineages were identified. Only clusters 412 and 442 had zero distance MLVA MtbC15-19 match to patterns stored in the MIRU-VNTR*plus* database. No other exact matches were identified for the study clusters.

Of the three largest clusters in Alberta, two spanned multiple provinces and one cluster was endemic to Alberta. When the data were further broken down by year of submission, the distribution of these predominant clusters in Alberta demonstrated a spike in 2014 which was an artifact, as sample submissions from 2012 and 2013 were delayed (Figure S1). Of the three largest clusters in British Columbia, two major clusters spanned multiple provinces and one cluster was endemic to British Columbia. When the data were further broken down by year of submission, the distribution of these predominant clusters showed spikes in various years demonstrating ongoing outbreaks with surges (Figure S2). The three largest clusters in Saskatchewan were also seen in other provinces. However, all were predominant in Saskatchewan. None of these clusters was endemic to Saskatchewan. When the data were further broken down by year of submission, the distribution of these predominant clusters showed ongoing outbreaks over multiple years with some surges in the 10-year study period (Figure S3). Of the largest three clusters in Manitoba, two clusters were also seen in other provinces and one was endemic to Manitoba. All three clusters remained predominant in Manitoba. When the data were analyzed by year of submission, cluster 2327 showed spikes of cases in 2009, 2013, and 2016, whereas the other two clusters appear to have flat-lined in recent years (Figure S4). Of the largest three clusters in Quebec, only one cluster was seen in another province. The largest two clusters were endemic to Quebec. When the data were further broken down by year of submission, cluster 2712 showed spikes in a couple of years. The other two clusters had low incidence rates and showed small resurgences over years (Figure S5). Of the largest three clusters in the Atlantic provinces, clusters 1952 and 1953 were seen only in Newfoundland and Labrador. Both of these clusters showed a spike after the year 2014. Cluster 52 was seen in multiple provinces (Figure S6). However, when investigated further, this cluster was *M. bovis* BCG isolated from disseminated BCG infections, so these do not provide evidence of transmission. One key issue is that future transmission events between provinces need to be better investigated via data collection and collaboration. A communication platform and real-time access to this data may overcome some of these issues.

There is an urgent need for case tracking using genotyping surveillance in the Canadian North; circulating tuberculosis strains are increasingly implicated in ongoing outbreaks that are difficult to resolve due to the population's increased mobility to neighboring communities [32]. For some predominant clusters that showed clonal transmission, MIRU genotyping was unable to resolve the chain of transmission in Nunavik (QC). For example, tuberculosis isolates collected from some select communities in Northern Canada may have 1–4 circulating MIRU-VNTR patterns that are closely related [6, 26]. The low resolution of MIRU-VNTR data is inadequate in some clusters to perform source tracking of clonal outbreaks. In such cases, a high-resolution genotyping test such as the WGS of *M. tuberculosis* [5] should be performed.

In recent years, public health laboratories have made major advancements by revolutionizing their diagnostic and surveillance capability [7, 23, 28, 31]. Using a universal genotyping method is a great step for tuberculosis control in Canada [8]. One major limitation is that the isolates were not all tested in real time for *M. tuberculosis* genotyping. When performing testing in real time, the submission statistics would accurately reflect the number of culture-positive cases per year per jurisdiction. The impact of this data would be powerful and could be used to improve outbreak detection [31]. A spiking trend may cause a cluster alert which could then be used to manage an upcoming outbreak. The housing of national data in one database whereby transmission of tuberculosis clusters between different jurisdictions within Canada can be observed and investigated in real time would be an added advantage. This 10-year data compilation highlights the importance of routine genotyping, investigation of clusters within the jurisdiction as well as across different jurisdictions, and watching a cluster trend over time. The real-time electronic access to this information shared between all jurisdictions would make future investigations faster and easier.

As a cluster may or may not represent an outbreak or recent transmission, a high-resolution genotyping method combined with epidemiological information could impact public health by reducing the amount of contact tracing. If these data were linked with cases, it would enhance national tuberculosis surveillance in Canada and genotyping results could be used to control tuberculosis at the provincial and federal levels [4, 6, 23]. Investigations are challenging for tuberculosis as reactivation can occur years after infection. The designation of cluster numbers based on genotyping data helps to inform tuberculosis transmission and is easier to communicate with nonlaboratory professionals. The future inclusion of surveillance information in the database would further enhance its capabilities. However, quality assessment of generated data, testing, and validation are all important factors. With data analysis focusing on clinical management and transmission tracking, economic benefits may be immensely valuable. The determination of a genetic relatedness metric generated from advanced methods, such as WGS data can better identify the chain of tuberculosis transmission in an outbreak, rapid identification of the source case, and thus help break the cycle of tuberculosis transmission [17].

## 5. Conclusion

Genotyping of bacteria helps confirm epidemiological connections in an outbreak and thus aids in public health investigations. Both genotyping and social network analyses complement each other by adding or removing cases in an outbreak [3, 4]. This makes an investigation more accurate and enables disease outbreaks to be tackled efficiently in real time. This remains true for investigations within a province, country, and globally. A genotyping method is highly effective when the method is rapid, technically easy, has high discrimination between clusters, and provides a user-friendly output that can be used to compare the results between laboratories nationally and internationally. MIRU-VNTR tuberculosis genotyping has proven itself to meet all the above criteria.

In the Canadian North, there is an urgent need for case tracking using high-resolution genotyping surveillance solutions due to several issues, such as population mobility being higher and genetic diversity of circulating tuberculosis strains being low. Identifying cases of reinfection versus relapse can have an important impact on cluster investigation [5–7]. The Health Canada Strategy, based on high-quality tuberculosis programming at the community and regional levels, echoes the Canadian Tuberculosis Standard defined tuberculosis control activities, such as early case finding, contact identification, and surveillance (data collection, analysis, and dissemination) [2, 7, 32, 33]. With newer methods and database advancements, public health impact through the use of routine genotyping in tuberculosis is increasingly evident [4, 6, 34, 35]. In conclusion, a combination of strong interjurisdictional networks for data-sharing and regular updates to genotyping methods can maximize the effectiveness of surveillance approaches and programs for infectious diseases.

## Figures and Tables

**Table 1:** Major Canadian MIRU-VNTR patterns and their closest MLVA, SpolDB4 ST, and phylogenetic tree matches against MTBC lineages contained in the MIRU-VNTRplus database.

ID	MLVA MtbC15-19	Top MLVA MtbC15-19 matches	Distance match	SpolDB4 ST match	Closest lineage match (ST)	Closest match from a phylogenetic tree^*∗*^
Cluster 412	115-44	115-44 113-44	0 0.41	19 19	EAI EAI	EAI
Cluster 442	113-44	113-44 115-44	0 0.41	19 19	EAI EAI	EAI
Cluster 2814	4063-15	143-62 82-15 83-15	0.21 0.25 0.25	(−) 50 287	X Haarlem Haarlem	TUR, X, Haarlem
Cluster 2425	NM-62	127-56 74-26 153-64	0.25 0.29 0.29	20 (−) 451	LAM Cameroon H37Rv	H37Rv, Cameroon
Cluster 1729	NM-15	140-15 133-15 139-60	0.13 0.17 0.17	34 34 71	S S S	S
Cluster 2327	21260-15	72-26 73-26 75-26	0.29 0.29 0.29	61 61 (−)	Cameroon Cameroon Cameroon	H37Rv, Cameroon
Cluster 1266	NM-1039	150-32 69-25 148-32	0.36 0.41 0.41	26 53 357	Delhi/CAS Ghana Delhi/CAS	Delhi/CAS
Cluster 2712	NM-15	92-15 142-15	0.17 0.21	47 119	Haarlem X	Haarlem
Cluster 2726	NM-15	84-28 92-15	0.167 0.167	1586 47	Haarlem Haarlem	Haarlem
Cluster 1952	NM-69	140-15 133-15 21-13	0.29 0.33 0.37	34 71 (−)	S S UgandaII	S

NM, no match; MTBC lineages contained in the MIRU-VNTRplus database include EAI, TUR, *X*, Haarlem, H37Rv, Cameroon, S, Delhi/CAS; ST, SpolDB4 strain type with missing STs in MIRU-VNTRplus represented by a hyphen sign (−) in this table. ^*∗*^Dendrogram is shown in supplementary material S7.

## Data Availability

The data used to support the findings of this study are included in the supplementary information.

## Supplementary Materials

Supplementary Materials Figure S1: Three largest clusters in Alberta. Figure S2: Three largest clusters in BC. Figure S3: Three largest clusters in Saskatchewan. Figure S4: Three largest clusters in Manitoba. Figure S5: Three largest clusters in Quebec. Figure S6: Three largest clusters in Atlantic provinces. Figure S7: Neighbor-joining tree generated from MIRU-VNTR*plus* with query cluster patterns shown highlighted.
