# Supplementary figures and images for: Compilation of 10 Years of MIRU-VNTR Data: Canadian National Tuberculosis Laboratory's Experience
Source: Can J Infect Dis Med Microbiol. 2022 Aug 22;2022:3505142. doi: 10.1155/2022/3505142 (PMC9424012; doi:10.1155/2022/3505142)

**Figure S1: Three largest clusters in Alberta**

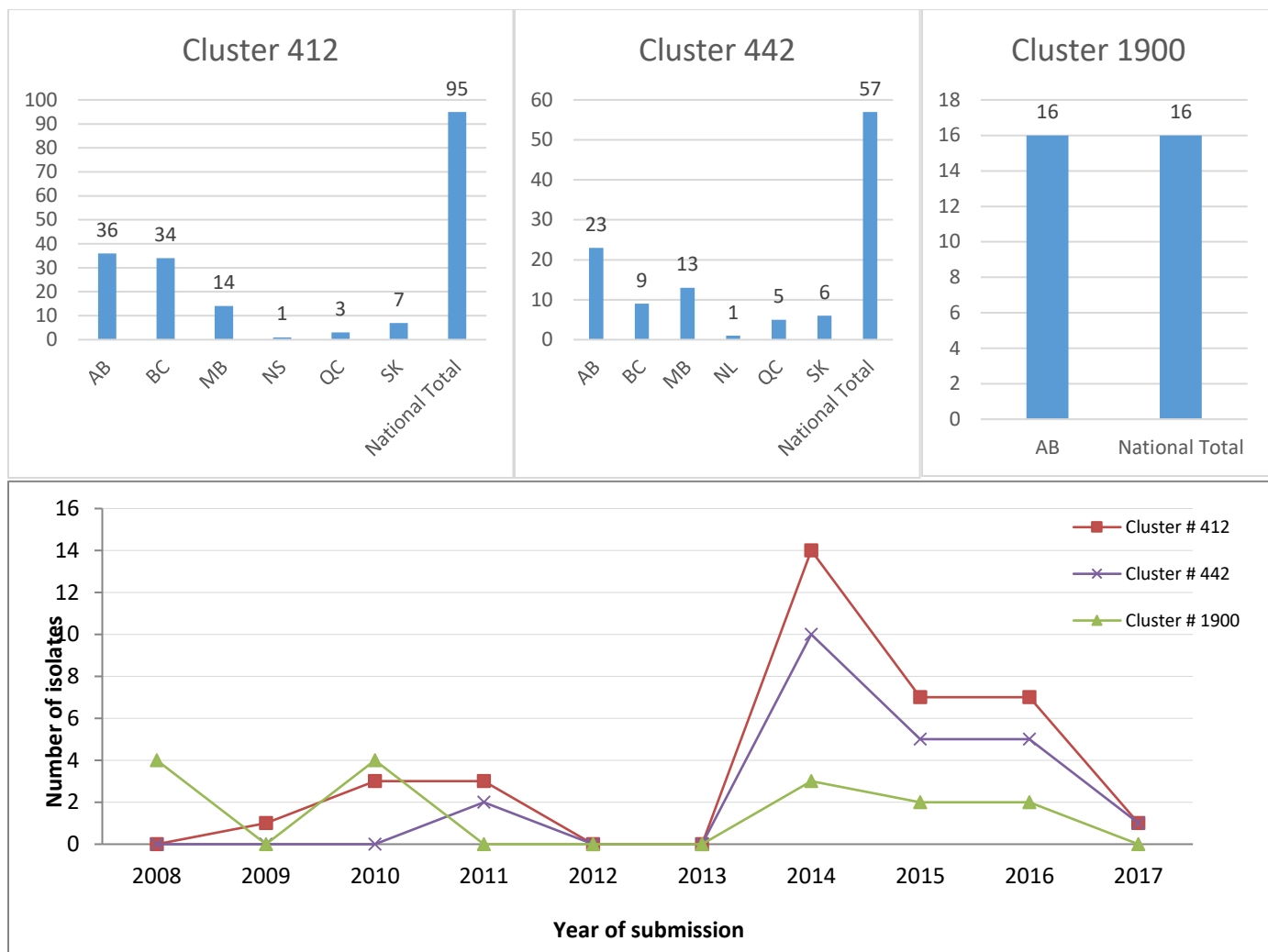

Supplement: Supplementary Materials — Figure S1: Three largest clusters in Alberta. Figure S2: Three largest clusters in BC. Figure S3: Three largest clusters in Saskatchewan. Figure S4: Three largest clusters in Manitoba. Figure S5: Three largest clusters in Quebec. Figure S6: Three largest clusters in Atlantic provinces. Figure S7: Neighbor-joining tree generated from MIRU-VNTRplus with query cluster patterns shown highlighted. [file 3505142.f1.zip › 3505142.f1/Figure S1.pdf]

**Figure S2: Three largest clusters in BC**

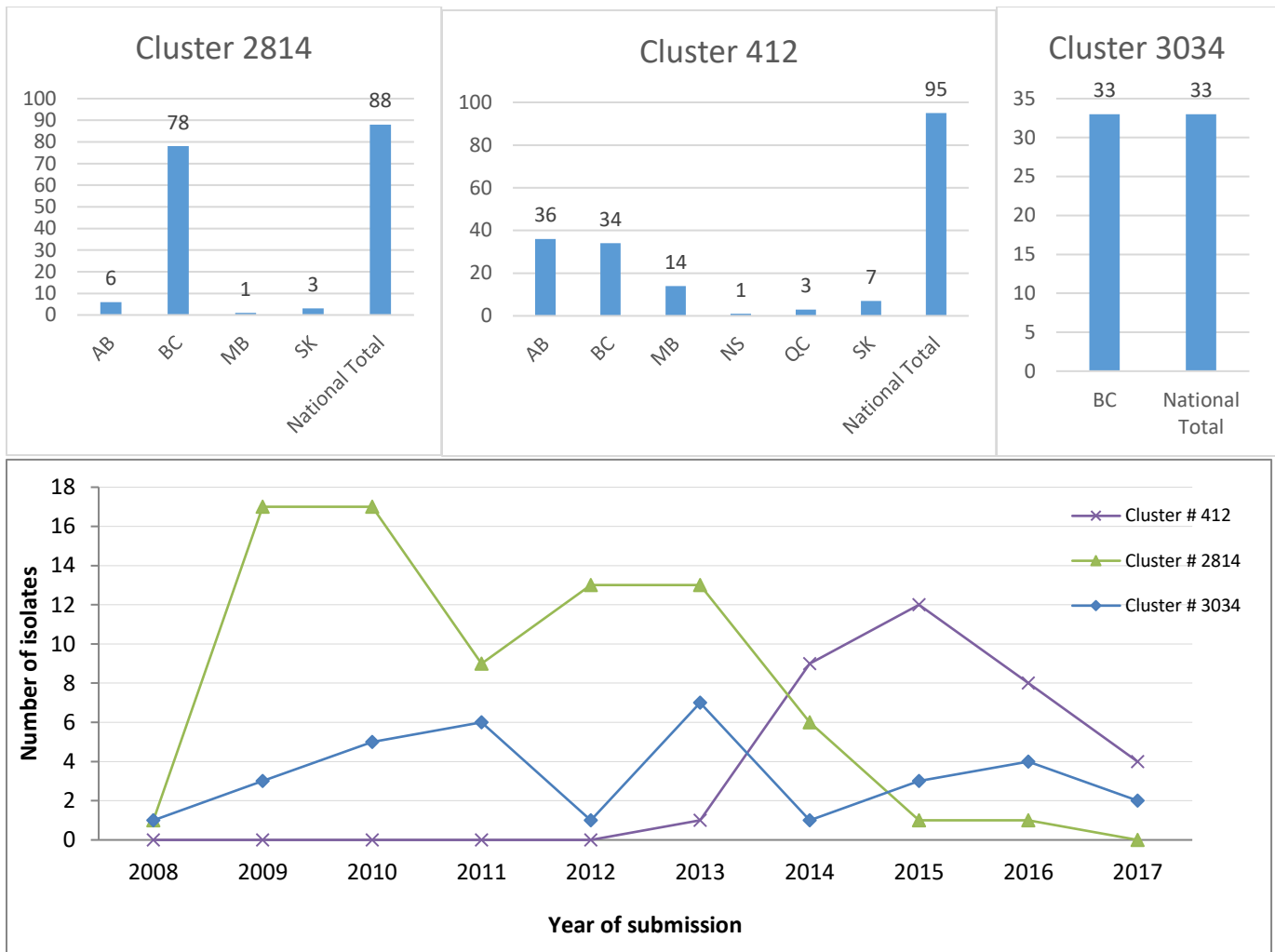

Supplement: Supplementary Materials — Figure S1: Three largest clusters in Alberta. Figure S2: Three largest clusters in BC. Figure S3: Three largest clusters in Saskatchewan. Figure S4: Three largest clusters in Manitoba. Figure S5: Three largest clusters in Quebec. Figure S6: Three largest clusters in Atlantic provinces. Figure S7: Neighbor-joining tree generated from MIRU-VNTRplus with query cluster patterns shown highlighted. [file 3505142.f1.zip › 3505142.f1/Figure S2.pdf]

**Figure S3: Three largest clusters in Saskatchewan**

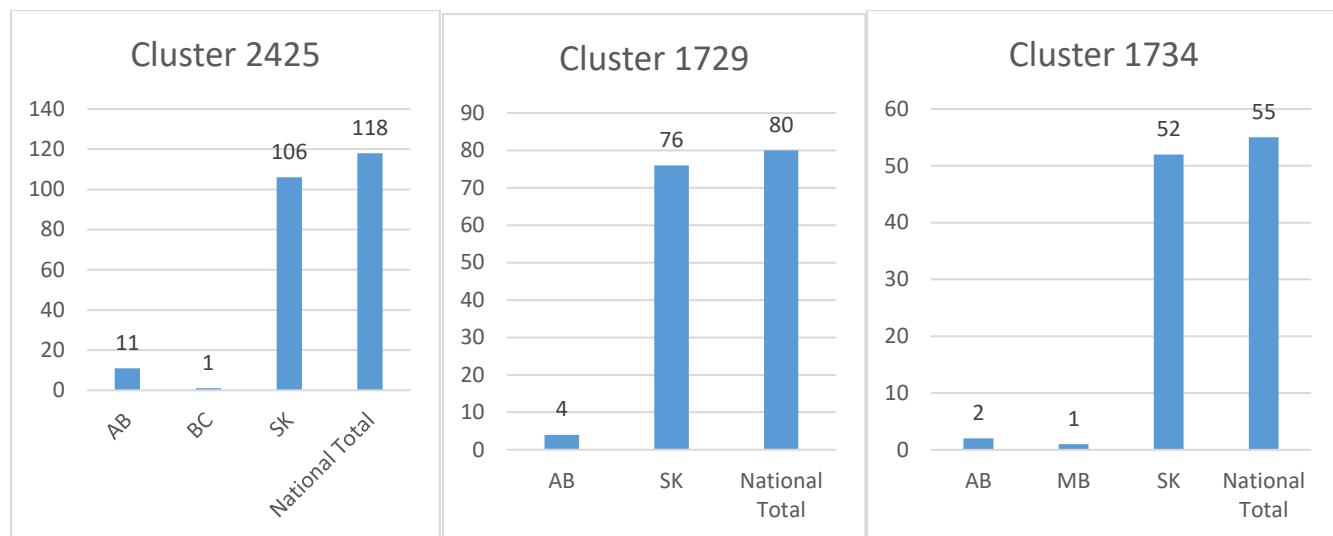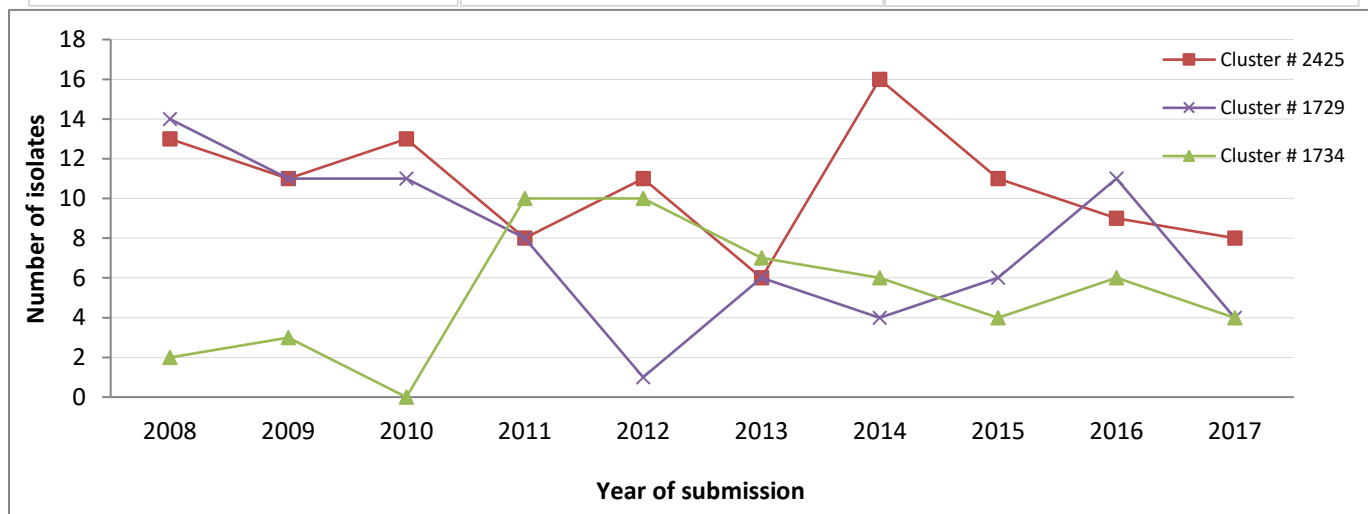

Supplement: Supplementary Materials — Figure S1: Three largest clusters in Alberta. Figure S2: Three largest clusters in BC. Figure S3: Three largest clusters in Saskatchewan. Figure S4: Three largest clusters in Manitoba. Figure S5: Three largest clusters in Quebec. Figure S6: Three largest clusters in Atlantic provinces. Figure S7: Neighbor-joining tree generated from MIRU-VNTRplus with query cluster patterns shown highlighted. [file 3505142.f1.zip › 3505142.f1/Figure S3.pdf]

**Figure S4: Three largest clusters in Manitoba**

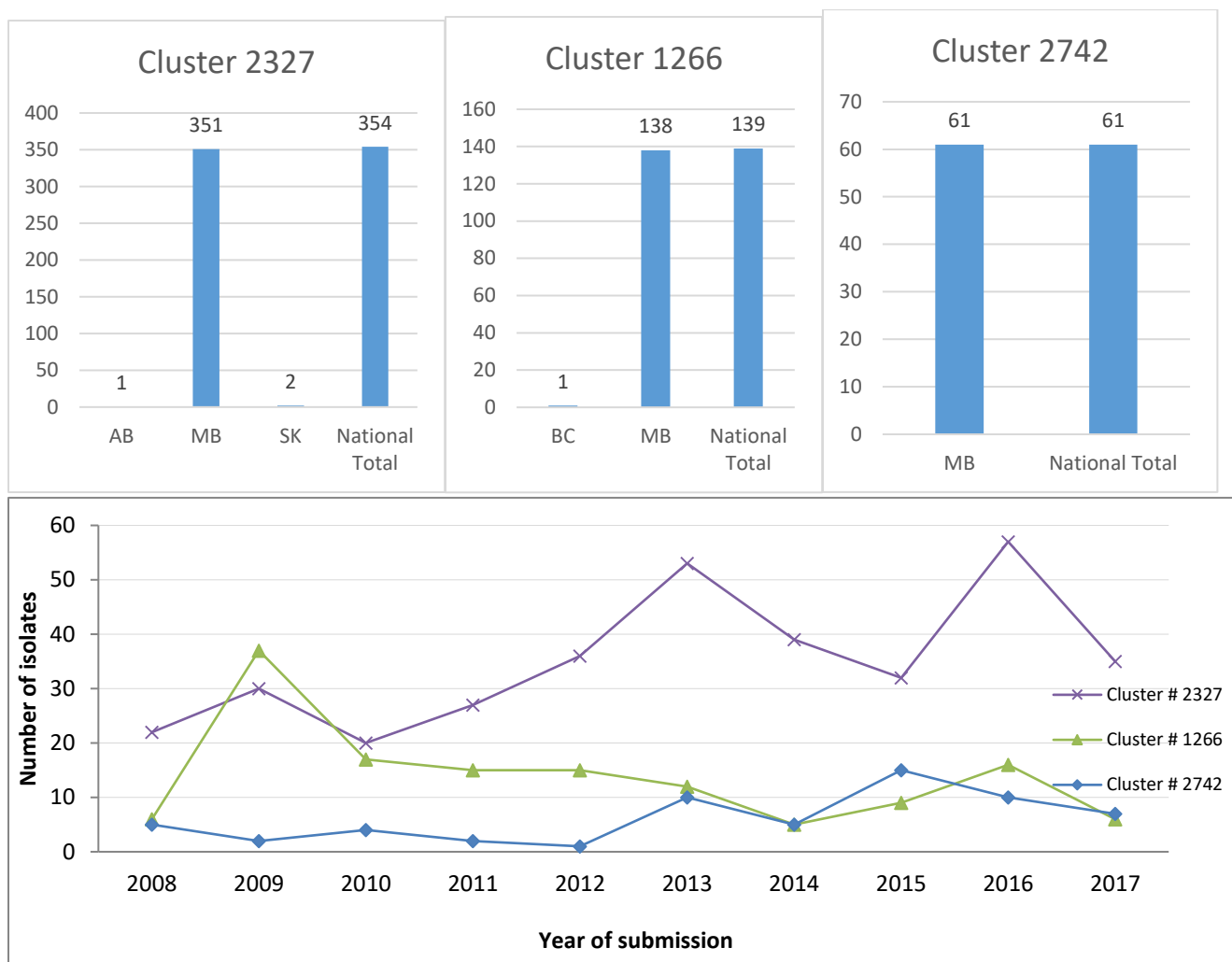

Supplement: Supplementary Materials — Figure S1: Three largest clusters in Alberta. Figure S2: Three largest clusters in BC. Figure S3: Three largest clusters in Saskatchewan. Figure S4: Three largest clusters in Manitoba. Figure S5: Three largest clusters in Quebec. Figure S6: Three largest clusters in Atlantic provinces. Figure S7: Neighbor-joining tree generated from MIRU-VNTRplus with query cluster patterns shown highlighted. [file 3505142.f1.zip › 3505142.f1/Figure S4.pdf]

**Figure S5: Three largest clusters in Quebec**

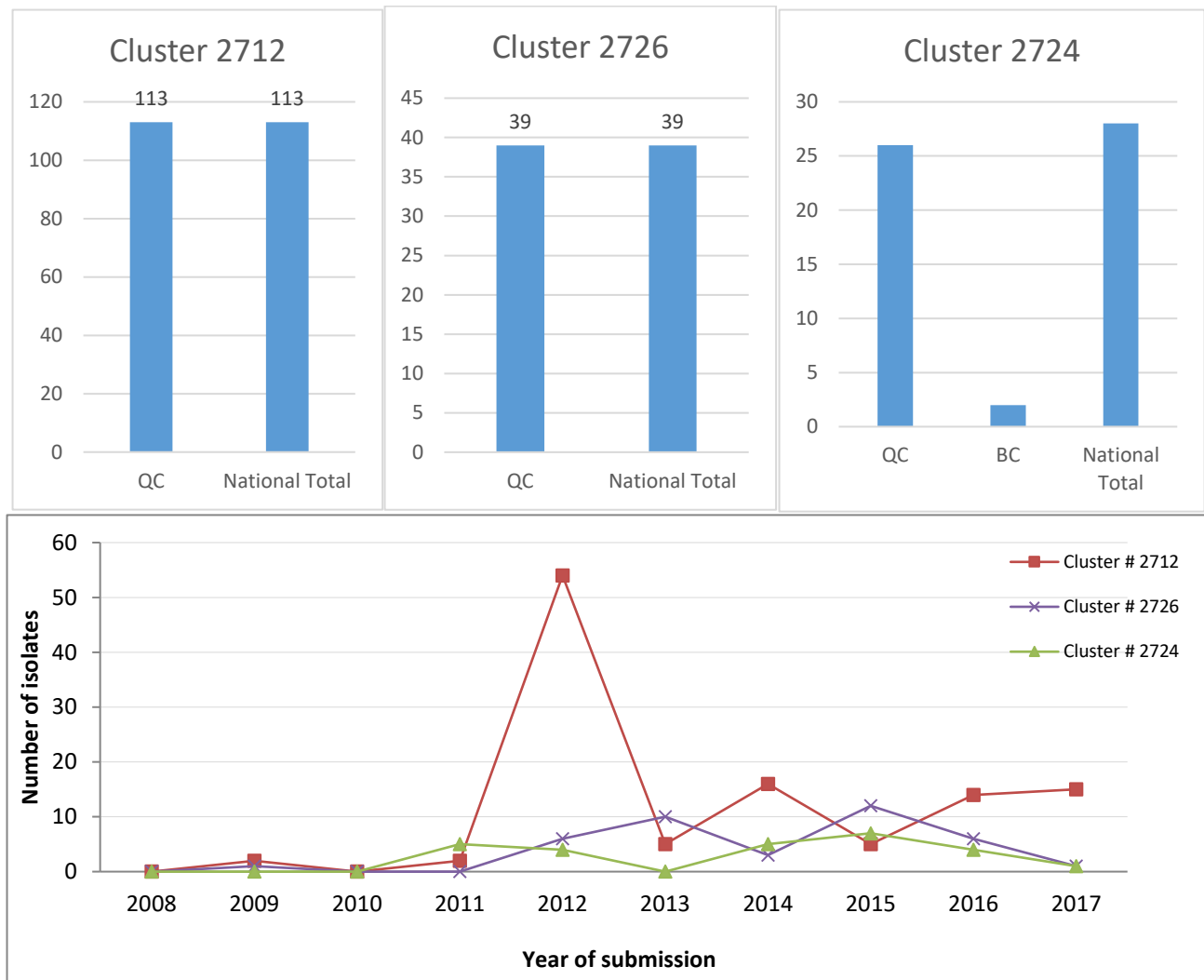

Supplement: Supplementary Materials — Figure S1: Three largest clusters in Alberta. Figure S2: Three largest clusters in BC. Figure S3: Three largest clusters in Saskatchewan. Figure S4: Three largest clusters in Manitoba. Figure S5: Three largest clusters in Quebec. Figure S6: Three largest clusters in Atlantic provinces. Figure S7: Neighbor-joining tree generated from MIRU-VNTRplus with query cluster patterns shown highlighted. [file 3505142.f1.zip › 3505142.f1/Figure S5.pdf]

**Figure S6: Three largest clusters in Atlantic provinces**

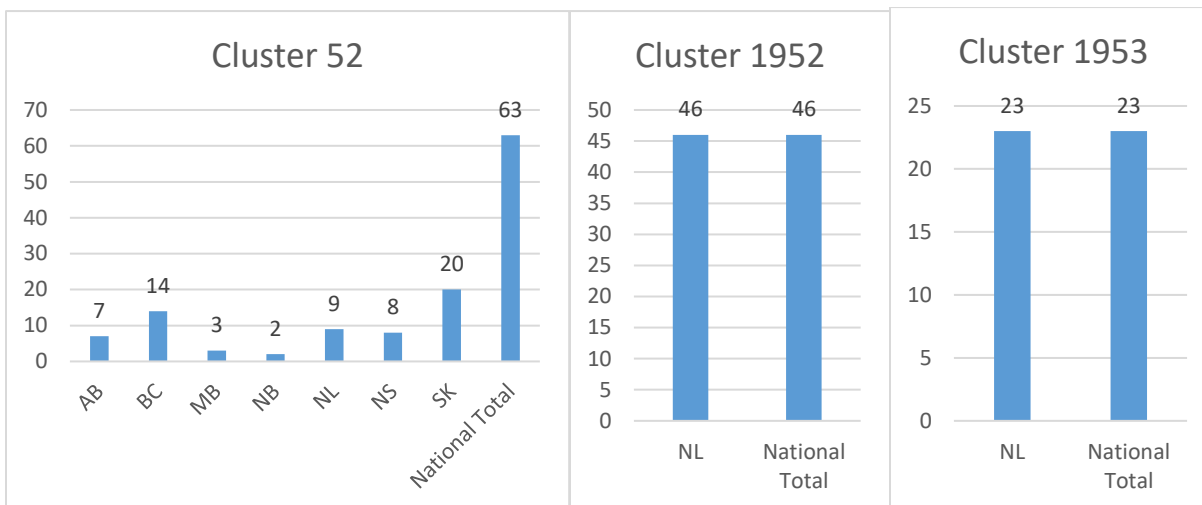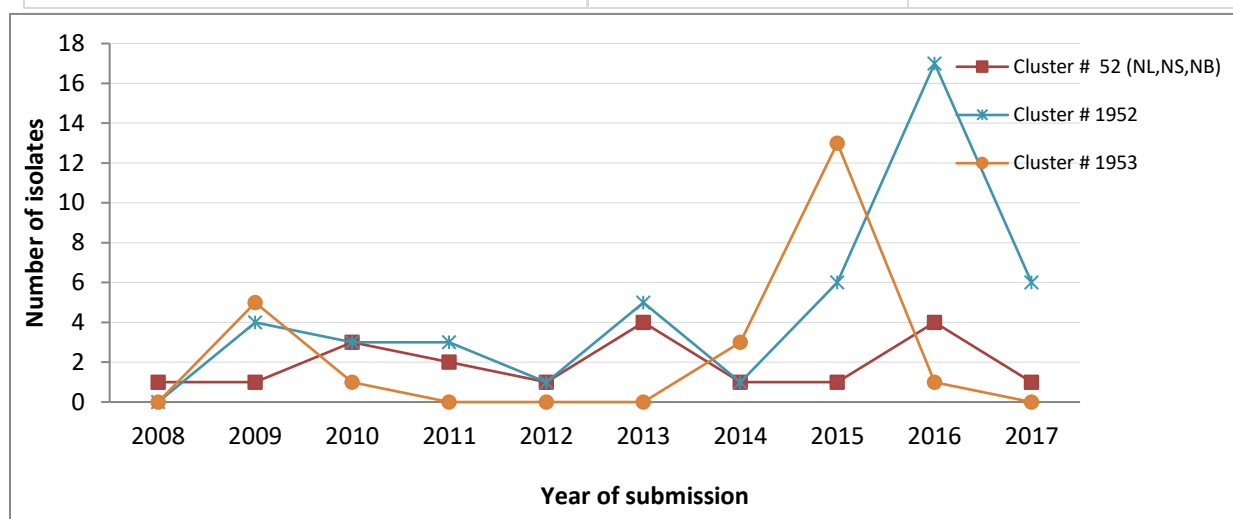

Supplement: Supplementary Materials — Figure S1: Three largest clusters in Alberta. Figure S2: Three largest clusters in BC. Figure S3: Three largest clusters in Saskatchewan. Figure S4: Three largest clusters in Manitoba. Figure S5: Three largest clusters in Quebec. Figure S6: Three largest clusters in Atlantic provinces. Figure S7: Neighbor-joining tree generated from MIRU-VNTRplus with query cluster patterns shown highlighted. [file 3505142.f1.zip › 3505142.f1/Figure S6.pdf]

Figure S7

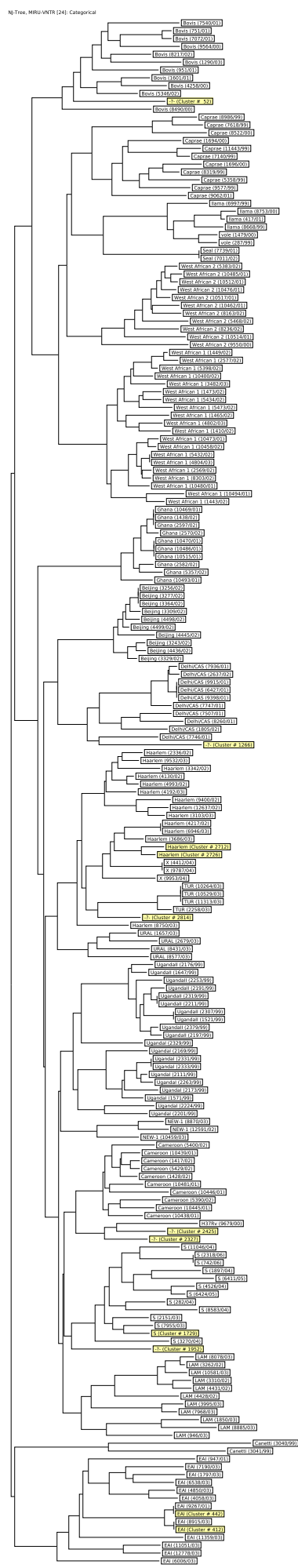

Supplement: Supplementary Materials — Figure S1: Three largest clusters in Alberta. Figure S2: Three largest clusters in BC. Figure S3: Three largest clusters in Saskatchewan. Figure S4: Three largest clusters in Manitoba. Figure S5: Three largest clusters in Quebec. Figure S6: Three largest clusters in Atlantic provinces. Figure S7: Neighbor-joining tree generated from MIRU-VNTRplus with query cluster patterns shown highlighted. [file 3505142.f1.zip › 3505142.f1/Figure S7 Neighbour-joining tree MIRUVNTRplus.pdf]
